# IRES-mediated translation of the carboxy-terminal domain of the horizontal cell specific connexin Cx55.5 in vivo and in vitro

**DOI:** 10.1186/1471-2199-9-52

**Published:** 2008-05-27

**Authors:** Mahboob Ul-Hussain, Georg Zoidl, Jan Klooster, Maarten Kamermans, Rolf Dermietzel

**Affiliations:** 1 Department of Neuroanatomy and Molecular Brain Research, Ruhr-University Bochum, University Street 150, D-44801 Bochum, Germany; 2 International Graduate School of Neuroscience (IGSN), Ruhr-University Bochum, D-44780 Bochum, Germany; 3 Research Unit Retinal Signal Processing, The Netherlands Institute for Neuroscience, Meibergdreef 47, 1105 BA Amsterdam, The Netherlands; 4 Department of Neurogenetics, Academic Medical Center, University of Amsterdam. Meibergdreef 9, 1105 AZ Amsterdam, The Netherlands

## Abstract

**Background:**

Changes of the interneuronal coupling mediated by electrical synapse proteins in response to light adaptation and receptive field shaping are a paramount feature in the photoreceptor/horizontal cell/bipolar cell (PRC/HC/BPC) complex of the outer retina. The regulation of these processes is not fully understood at the molecular level but they may require information transfer to the nucleus by locally generated messengers. Electrical synapse proteins may comprise a feasible molecular determinant in such an information-laden signalling pathway.

**Results:**

Connexin55.5 (Cx55.5) is a connexin with horizontal cell-restricted expression in zebrafish accumulating at dendritic sites within the PRC/HC/BPC complex in form of hemichannels where light-dependent plasticity occurs. Here we provide evidence for the generation of a carboxy-terminal domain of Cx55.5. The protein product is translated from the Cx55.5 mRNA by internal translation initiation from an in-frame ATG codon involving a putative internal ribosome entry site (IRES) element localized in the coding region of Cx55.5. This protein product resembling an 11 kDa domain of Cx55.5 is partially located in the nucleus *in vivo *and *in vitro*.

**Conclusion:**

Our results demonstrate the generation of a second protein from the coding region of Cx55.5 by an IRES mediated process. The nuclear occurrence of a fraction of this protein provides first evidence that this electrical synapse protein may participate in a putative cytoplasmic to nuclear signal transfer. This suggests that Cx55.5 could be involved in gene regulation making structural plasticity at the PRC/HC/BPC complex feasible.

## Background

Direct communication via gap junctions between cells is important for coordinated cellular activity. Connexins play a central role in this biological function and contribute to tissue homeostasis and electrical coupling by forming communicating channels between adjacent cells. In general, the significance of connexin expression has been attributed to gap junction coupling. However, recent evidence suggests that connexins may play other roles than being the integral part of gap junction channels. In fact, connexins and/or processed connexin fragments may influence important biological functions like regulation of cell growth [1-3] and resistance to cell death [4] by mechanisms that do not require gap junction communication [5-8] but necessitate cytoplasm to nucleus signalling by locally generated messengers.

In brain tissues interneuronal signalling is conveyed by chemical and electrical synapses, the latter being formed by gap junctions. Extensive data exists on the nature of locally generated messengers which target to the nucleus serving important function for activity-dependent control of neuronal gene expression during chemical signalling transmission [9-11]. Evidence for mechanisms that may play a similar role is entirely missing for electrical synapses.

We chose the photoreceptor/horizontal cell/bipolar cell (PRC/HC/BPC) complex of the retina to screen for such mechanism for the following reasons: (i) The PRC/HC/BPC complex is endowed with connexins either in form of hemichannels and/or of paired gap junctions [12]. (ii) The PRC/HC/BPC complex exhibits a remarkable restructuring in response to ambient light exposure, and can be regarded as a model for long-term activity-dependent electrical synapse plasticity [13-15]. (iii) HCs are unique insofar as they reveal a highly restricted pattern of connexin expression. Mouse HCs express Cx57 the orthologue of the human Cx59 [16]. In zebrafish the expression of two related connexins has been described: Cx52.6 and Cx55.5 [17,18] with the latter accumulating in HC dendrites which are involved in the activity dependent plasticity of the PRC/HC/BPC complex [17].

All connexin isoforms are presumed to have similar topology, which has been deduced from limited proteolysis and the application of site directed antibodies [19]. The NH_2_-terminal and the COOH-terminal domain are localized in the cytoplasm and are connected by four transmembrane domains, two extracellular loops and a cytoplasmic loop. Recent evidence indicates that the carboxy-terminus of one of the most abundant connexins (Cx43) may be involved in gene regulation by either interacting with growth regulators [20,21] or nuclear-translocation of processed carboxy-terminal domains [22].

The coding region of the zebrafish Cx55.5 lies in a single main exon consisting of 1497 bp [17] located downstream of two promoters [23]. It shares the same topology with other connexins and exhibits an exceptionally long COOH-terminal tail of ~288 amino acids characterized by an unusual clustering of serine residues. Thus Cx55.5 provides a likely candidate to look for activity-dependent processing of signalling domains. Evidence that some connexins are regulated at the posttranscriptional level initially derived from studies with Cx26, Cx32 or Cx43. The 5'-UTRs of these connexins contain an internal ribosome entry site (IRES), enabling translation in a cap-independent manner [24-26]. Additionally, it has been demonstrated that the Xenopus Cx41 5'-UTR contains three upstream open reading frames (uORFs) that significantly repress translation in young embryos [27].

In the present report, we provide evidence for a unique way of an internal expression of a carboxy-terminal domain of Cx55.5, mediated by a putative IRES element present within the coding region. Activation of this IRES element elicits the processing of an 11 kDa carboxy-terminal fragment (p11-CT) which could be detected in the cytoplasm and the nucleus of the retina *in vivo *and in heterologous expression systems *in vitro *suggesting a cytoplasm to nucleus signalling mechanism through a messenger generated from an electrical synapse protein.

## Results

### Cx55.5 immunoreactivity in the nucleus of horizontal cells and identification of a candidate protein

**Figure 1:**
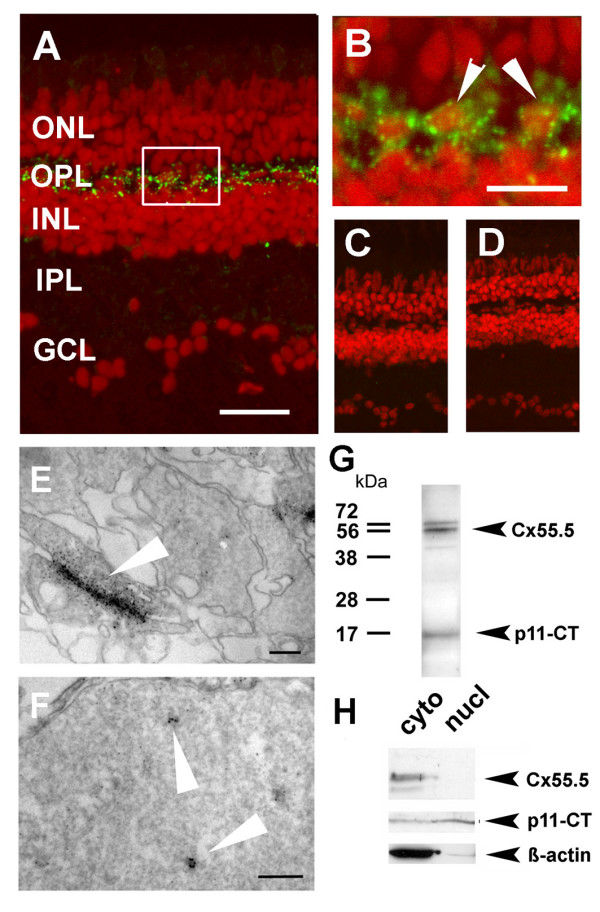
**Localization of Cx55.5 immunoreactivity in the nucleus of horizontal cells**. A) Confocal imaging using z-stack recordings reproducibly showed that Cx55.5 (green fluorescence) was exclusively expressed in horizontal cells of the outer retina. Scale bar: 100 μm B) The higher magnification of the region of interest (frame in A) provided evidence for small clusters of Cx55.5 immunoreactivity inside the nuclei of HCs (see arrowheads). Scale bar: 20 μm. Antibody controls, C) omitting the primary antibody and D) blocking of the primary antibody by pre-absorption with 1 μg GST-Cx55.5 fusion protein. Nuclei were stained with Sytox Orange (Invitrogen, Karlsruhe, Germany). In order to exclude the possibility that false positive fluorescent emission was recorded from out of focus planes electron microscopical immunohistochemistry of adult retina was performed. Gap junction plaques (arrow) between HCs as shown in E) resemble characteristic membrane associated structures detected with the Cx55.5 antibody, whereas the formation of small clusters (arrows) within the nucleus as shown in F) was typical for nuclear Cx55.5 immunoreactivity. The localization and number of clusters in a single section as shown in the inset was variable but did not exceed N = 3. Note that Cx55.5 immunoreactivity was never found outside the HC layer. Scale bars: 400 nm (E), 300 nm (F). (G) Western blot analysis of total protein extracts isolated from adult retina indicates a doublet of bands at the position 56 kDa and a low molecular weight band at the position 16 kDa. H) Western blot analysis of protein extracts isolated by subcellular fractionation of the adult retina (cyto = cytosolic fraction, nucl = nuclear fraction). The full length Cx55.5 protein product was enriched in the cytoplasmic fraction which is composed of soluble cytosolic proteins and membrane proteins (upper lanes). The 16 kDa protein product was found in the cytosolic and nuclear fraction (middle lanes). The lower blot depicts the β-actin control. Note the faint β-actin band in the nuclear fraction indicating minimal contamination with cytoplasmic remnants. The results shown derived from two independent western blot runs using the same samples (upper and lower lanes: 10% SDS-PAGE; middle lane; 15% SDS-PAGE).

We have recently reported that Cx55.5 is exclusively expressed in HCs of the zebrafish retina. This was demonstrated using transgenic fish carrying a Cx55.5-promoter driven EYFP transgene and by immunocytochemistry with affinity purified polyclonal antibodies directed against the carboxy-terminal (CT) domain of the Cx55.5 protein [12]. In addition to the reported cytoplasmic and membrane-bound localization of Cx55.5 found in HCs of the outer retina as shown in Fig. 1A and the Additional file 1, we reproducibly observed a low abundant but specific immunoreactivity in the nuclei of HCs. At higher magnification nuclear signals appeared in form of small fluorescent spots distinct from the strong reactivity localized to the cytoplasm and to the plasma membrane (Fig. 1B). Such immunoreactivity was not observed outside the HC layer. Control experiments using peptide blocking (Fig. 1C) or omitting the primary antibody (Fig. 1D) supported the specificity of our detection approach. In addition we reconstructed Z-stack recordings from confocal images using the orthogonal view function of the LSM510 Meta software (see under methods). Since we were unable to exclude the possibility that the fluorescent signals observed may derive from bleed through fluorescence from out of focus planes we performed immuno-electron microscopy to confirm the specificity of our initial observation. As shown at the ultrastructural level typical membrane-associated Cx55.5 immunoreactivity was frequently found in form of gap junctions between HCs (Fig. 1E). Immunoreactivity found exclusively in the nuclei of HCs appeared in form of small and patchy aggregates (Fig. 1F). Cx55.5 immunoreactivity was never observed in cells outside the horizontal cell layer. In order to prove the identity of this protein we analyzed retina protein extracts by western blots (Fig. 1G). The reactivity of our antibody with the full length Cx55.5 protein was exclusive for the retina which is consistent with our previously reported observations [12]. In the retina the Cx55.5 antibody reacted specifically with a protein of a molecular weight of ~56 kDa. In addition to the full length 56 kDa protein a small protein product migrating at ~16 kDa was reproducibly detected (Fig. 1G). A yet uncharacterized higher molecular weight band may resemble a protein complex including the 56 kDa and 16 kDa protein products.

Since the antibody applied was generated by immunization with a fusion protein comprising the entire Cx55.5 CT domain (nt629–nt1497) it was reasonable to speculate that the ~16 kDa protein derived from the CT of Cx55.5, and may comprise the source for the nuclear reactivity. This assumption was experimentally confirmed using fractionated protein extracts deriving from the retina of adult fish (Fig. 1H). As expected the full length Cx55.5 protein product resembling an integral membrane protein was restricted to the cytoplasmic cell fraction composed of soluble and membrane proteins (upper lane). The ~16 kDa protein product was found in the cytoplasmic and nuclear extract (middle lane). Note that β-actin is depleted in the nuclear fraction as expected (bottom lane). Thus, the ~16 kDa fragment fulfilled the criteria as candidate protein for the Cx55.5 immunoreactivity observed by the immunohistochemical localization.

### Full length Cx55.5 and a portion of its carboxy-terminal domain are co-translated

**Figure 2:**
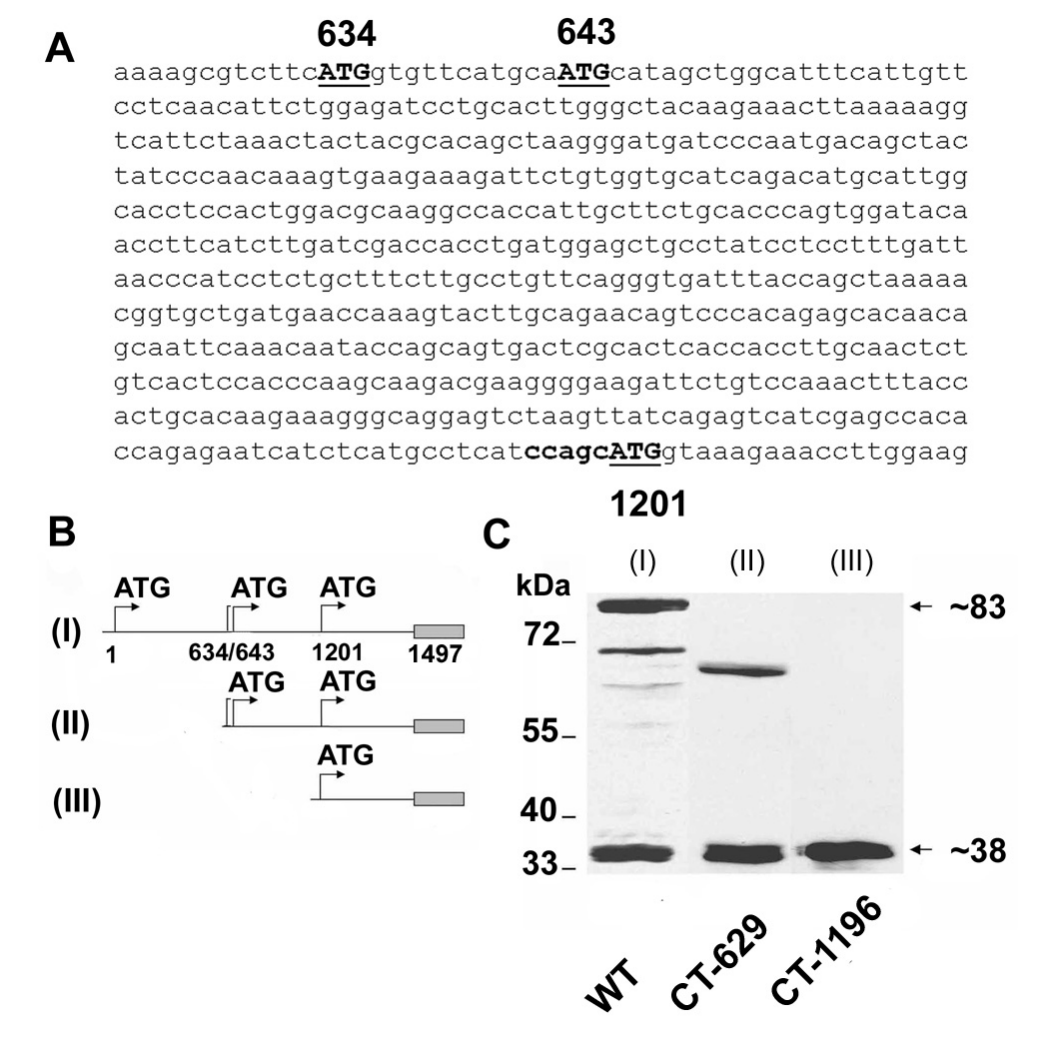
**Simultaneous expression of Cx55.5 and its carboxy-terminal domain**. A) Nucleotide sequence of the 5' end of the carboxy-terminal domain, with in-frame ATG codons at nucleotide positions 634, 643 and 1201 shown in bold. B) Schematic representation of EGFP-fusion protein constructs of full length Cx55.5 (WT, I; nt1 to nt1497), carboxy terminal domain of Cx55.5 (II; CT-629; nt629 to nt1497) and 3' half of carboxy-terminal domain (III; CT-1196; nt1196 to nt1497). C) Western blot analysis of transiently transfected N2A cells with EGFP fusion constructs: (lane I) full length Cx55.5, (lane II) full length carboxy-terminal domain (CT-629), (lane III) 3' half of the carboxy-terminal domain (CT-1196). Note that in lane I (full length Cx55.5 construct), besides 83 kDa and 38 kDa fusion protein bands, a few putative N-terminally truncated Cx55.5 protein bands are also visible.

One of several questions raised by our *in vivo *results was, whether a specific molecular mechanism is responsible for the generation of the ~16 kDa protein. In order to identify this mechanism, we analyzed the Cx55.5 coding sequence for alternative protein coding regions and identified several in frame ATG codons. In the CT domain three in frame ATGs were present starting at nt634, nt643 and nt1201 (Fig. 2A). Although the calculated molecular weight (~11 kDa) of the latter protein product (starting from nt1201) was lower than the experimentally determined ~16 kDa found by western blot it appeared possible on the basis of the start codon proceeded by a nearly perfect Kozak sequence (**ccagc**ATGg) that the candidate protein was translated from the in frame ATG codon at nucleotide position 1201. This hypothesis was tested *in vitro*. We engineered a fusion construct of the coding region of Cx55.5 with the enhanced green fluorescent protein (EGFP) using the pEGFP-N3 vector. After transient transfection into N2A cells, a whole cell extract was prepared 48 hours post transfection and subjected to western blot analysis. The Cx55.5-EGFP fusion protein with a calculated molecular weight of 82.5 kDa (Cx55.5 + 27 kDa for EGFP) was detected with a monoclonal anti-GFP antibody. Repeatedly higher mobility bands became apparent aside of the expected protein including a fusion protein band of ~38 kDa (11 kDa + 27 kDa EGFP; Fig. 2C, lane I). To further confirm that this protein is indeed derived from the CT portion of Cx55.5, we made two additional fusion proteins, one of which corresponds to the full length CT (CT-629; nt629 to nt1497), having its own in-frame ATG codon and a second construct that starts from nt1196 running to nt1497 (CT-1196; having the ATG codon at position 1201; Fig. 2B). Transient transfections into N2A cells and subsequent Western blot detection with the anti-GFP antibody showed a band at ~60 kDa (33 kDa CT-domain + 27 kDa EGFP) corresponding to the full length CT and an additional prominent band at ~38 kDa (Fig. 2C, lane II). The DNA construct with the Cx55.5 sequence from nt1196 to nt1497 showed the expected fusion protein band of ~38 kDa (Fig. 2C, lane III). The expression of the ~38 kDa fusion protein (in the following termed p11-CT referring to the calculated putative molecular weight of the Cx55.5-CT domain) fits the criteria of internal translation from an in-frame ATG codon at nt1201 in the coding region of Cx55.5.

### The carboxy-terminal protein p11-CT is translated from the Cx55.5 transcript by internal translation

**Figure 3:**
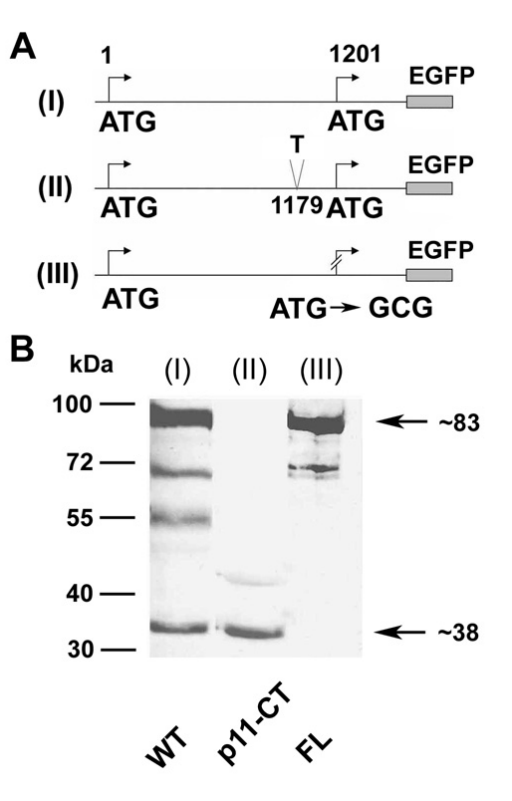
**A part of the carboxy-terminal domain of Cx55.5 is translated from an internal translation site within the coding region of Cx55.5**: Schematic view of wild type full length WT (I), frameshift mutated (position 1179) full length, p11-CT (II), and in-frame ATG replaced by GCG (position 1201) of full length Cx55.5 construct, FL (III). The frameshift mutation (II) leads to a premature stop codon at nucleotide position 1227. The carboxy-terminus of Cx55.5 is truncated by 90 amino acids and 15 amino acids are altered after the added "T" at nucleotide position 1179. B) Western blot of transiently transfected N2A cells: (lane I) Wild type (WT), (lane II) frameshift mutated p11-CT, and (lane III) ATG replaced by GCG of full length Cx55.5 (FL).

To elucidate the molecular mechanism responsible for the expression of the p11-CT protein, we introduced a frame shift mutation at nt1179 in the Cx55.5 coding region. This construct was transiently transfected in N2A cells. Western blot detection using the anti-GFP antibody showed that by creating the frame shift, translation of full length Cx55.5 was completely abolished when compared with the non-mutated full length Cx55.5 (Fig. 3, lane I) while the p11-CT protein can be still detected (Fig. 3, lane II). The disappearance of the full length protein and the persistent expression of p11-CT is a clear indication that a cleavage mechanism cannot be responsible for the generation of this carboxy-terminal protein. In fact, the above results supported our idea that the p11-CT protein is translated from the in frame ATG codon at nt1201. To test this hypothesis, we changed the in frame ATG (nt1201) to GCG as shown in Fig. 3A and expressed the mutation in N2A cells. Under this condition, immunoblot detection using anti-GFP antibody showed the presence of full length Cx55.5, while the expression of the p11-CT protein was completely abolished (Fig. 3, lane III).

### An IRES element in the coding region of Cx55.5 is responsible for the expression of the p11-CT protein

**Figure 4:**
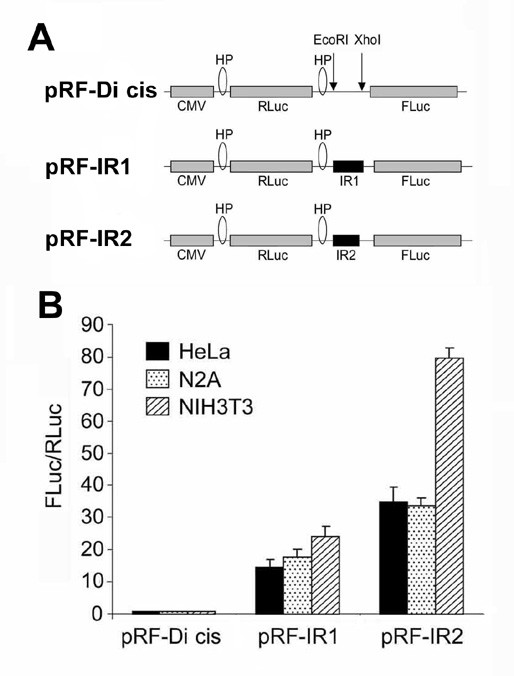
**Identification of an IRES element in the coding region of Cx55.5 using Di-cis vectors**. A) Schematic map of the pRF-Di cis vector having *Renilla *luciferase (RLuc) as the first cistron and *Firefly *luciferase (FLuc) as the downstream cistron, with two stable stem-loops or hairpins (HP), one at the 5' end and another at 3' end of *Renilla *luciferase gene. pRF-IR1 construct carrying the coding region (from nt631 to nt1200) of the CT in the intercistronic region, and pRF-IR2 construct with the coding region (from nt631 to nt990) of the CT in the intercistronic region. B) IRES activity of the above three constructs in N2A, HeLa and NIH3T3 cells. The IRES activity is represented as ratio of *Firefly *to *Renilla *luciferase activity (FLuc/RLuc) with the activity of the control vector, pRF-Di cis set is at 1. Each construct was tested five times and each experiment was done in triplicate. Data are expressed as mean ± SEM.

Next, we tested the possibility that a fragment of the coding region of the carboxy-terminus from nt631 to nt1200 contains an internal ribosome entry site (IRES). A 569 nt fragment ahead of the in-frame ATG start codon at nt1201 was subcloned into the inter-cistronic region of the di-cistronic vector pRF-Di-cis to generate pRF-IR1. The pRF-IR1 plasmid (Fig. 4A) and the control pRF-Di-cis vector (Fig. 4A) were transiently transfected into N2A cells. *Renilla *(RLuc) and *Firefly *luciferase (FLuc) activity were measured 48 hours post transfection. Luciferase activity readings depicted that the fragment was able to enhance the expression of the downstream located *Firefly *luciferase cistron by ~15 fold as compared to the control vector pRF-Di-cis (Fig. 4B). The pRF-IR1 construct was further transiently transfected into HeLa and NIH3T3 cell lines to test whether this putative IRES element is also active in other cell lines. 48 hours after transfection, luciferase activity was almost similar in HeLa and N2A cells, while in NIH3T3 cells the activity was increased ~25 fold compared to the control pRF-Di-cis vector (Fig. 4B).

To further delineate the putative IRES element, the pRF-IR1 construct was truncated by removing a ~200 bp fragment from the 3'end of the pRF-IR1 construct. This new construct pRF-IR2 (Fig. 4A) with a shortened Cx55.5-CT domain (nt631 to nt990) along with the construct carrying the entire fragment (pRF-IR1) and the control pRF-Di-cis vector were transiently transfected into N2A, HeLa and NIH3T3 cells. Subsequent luciferase activity determination indicated a substantial overall increase of IRES activity. The increase over control levels was ~35 fold in HeLa, ~34 fold in N2A, and ~77 fold in NIH3T3 cells (Fig. 4B). This observation indicates that the DNA sequence immediately upstream of the in frame ATG codon (nt1201) exhibits a regulative function on the IRES activity.

### Increased expression of the second cistron in the Di-cistronic assay is due to IRES activity and not a cryptic promoter

**Figure 5:**
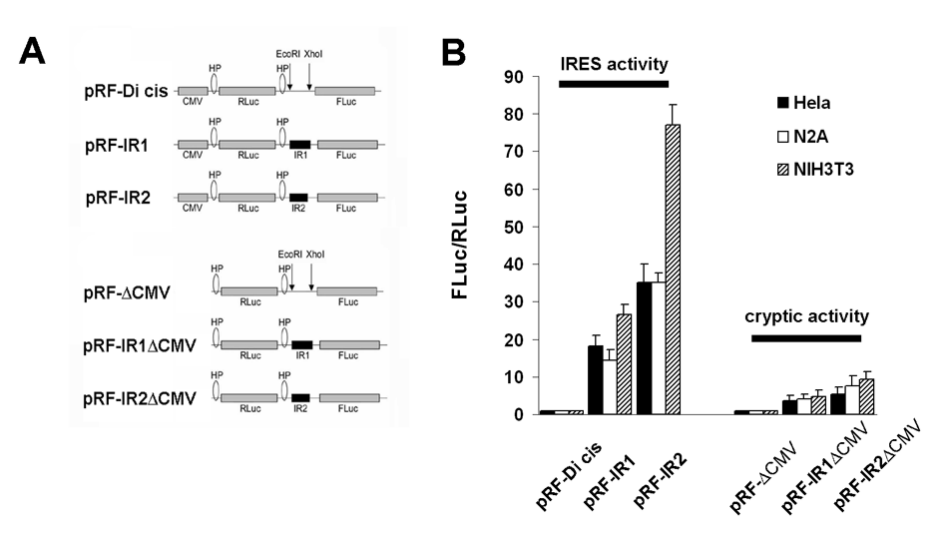
**IRES activity versus cryptic promoter activity of the coding region of Cx55.5**. A) Schematic presentation of the different Di-cis constructs and the respective promoterless Di-cis constructs (see Additional File 4 for further explanation). B) IRES activity and cryptic activity of the above constructs in N2A, HeLa and NIH3T3 cells. The IRES activity is represented as the ratio of *Firefly *to *Renilla *luciferase (FLuc/RLuc) with the activity of the control vector, pRF-Di cis, set at "1". Each construct was tested three times and each experiment was done in triplicate. Data are expressed as mean ± SEM

To rule out the possibility that the increased expression of the second cistron in the Di-cistronic assay is due to a cryptic promoter activity, promoterless Di-cistronic constructs pRFΔCMV, pRF-IR1ΔCMV and pRF-IR2ΔCMV were prepared from pRF-Di-cis, pRF-IR1 and pRF-IR2 constructs by removing the CMV promoter (Fig. 5A). Each promoterless Di-cis construct was transiently transfected into N2A, HeLa and NIH3T3 cells. The ratio of *Firefly *to *Renilla *luciferase activity decreased substantially under control conditions as compared to the 34 fold to 77 fold increase in the IRES containing vectors suggesting that the IRES element and not a cryptic promoter activity is responsible for the increased expression (Fig. 5B, C). The minimal change of the expression ratio of the promoterless Di-cis IRES vector can be explained by some leaky transcription initiated from the vector backbone.

**Figure 6:**
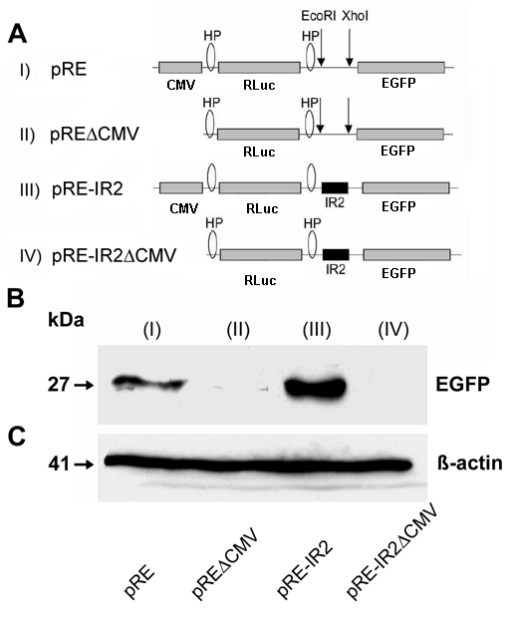
**Confirmation of the activity of the IRES element by western blot analysis**. A) Schematic representation of the Di-cis vectors with EGFP as second cistron. (I) indicates pRE as control vector, (II) pREΔCMV with the corresponding promoterless control vector, (III) shows the pRE-IR2 with IRES element IR2 (nt631 to nt990) in the inter-cistronic region, and (IV) the corresponding promoterless construct (pRE-IR2ΔCMV). B) Western blot of the expression product of the Di-cis constructs transiently transfected in N2A cells: The blots show the increased expression of the downstream EGFP cistron mediated by the IR2 element (III). C) Western blot of β-actin as loading control.

These findings were confirmed by western blot analysis. For this purpose pRF-Di-cis, and pRF-IR2 vectors were modified by removing the *Firefly *luciferase cistron and replacing it with the EGFP gene in the position of the second cistron (Fig. 6A). All constructs were transiently transfected into N2A cells and analyzed 48 hours after transfection. Immunodetection with anti-GFP antibodies showed an enhanced expression of EGFP in the presence of the IRES element (pRE-IR2; lane III) as compared to control vector pRE (lane I). The promoterless control vector, pRE-ΔCMV (lane II) showed a faint expression of EGFP which lead credence to our observation of some leaky transcription. Promoterless IRES vector, pRE-IR2ΔCMV (lane IV) showed no expression at all (Fig. 6B). Equal protein loading was confirmed by immunodetection of β-actin (Fig. 6C).

### The p11-CT protein is not expressed from a monocistronic mRNA

**Figure 7:**
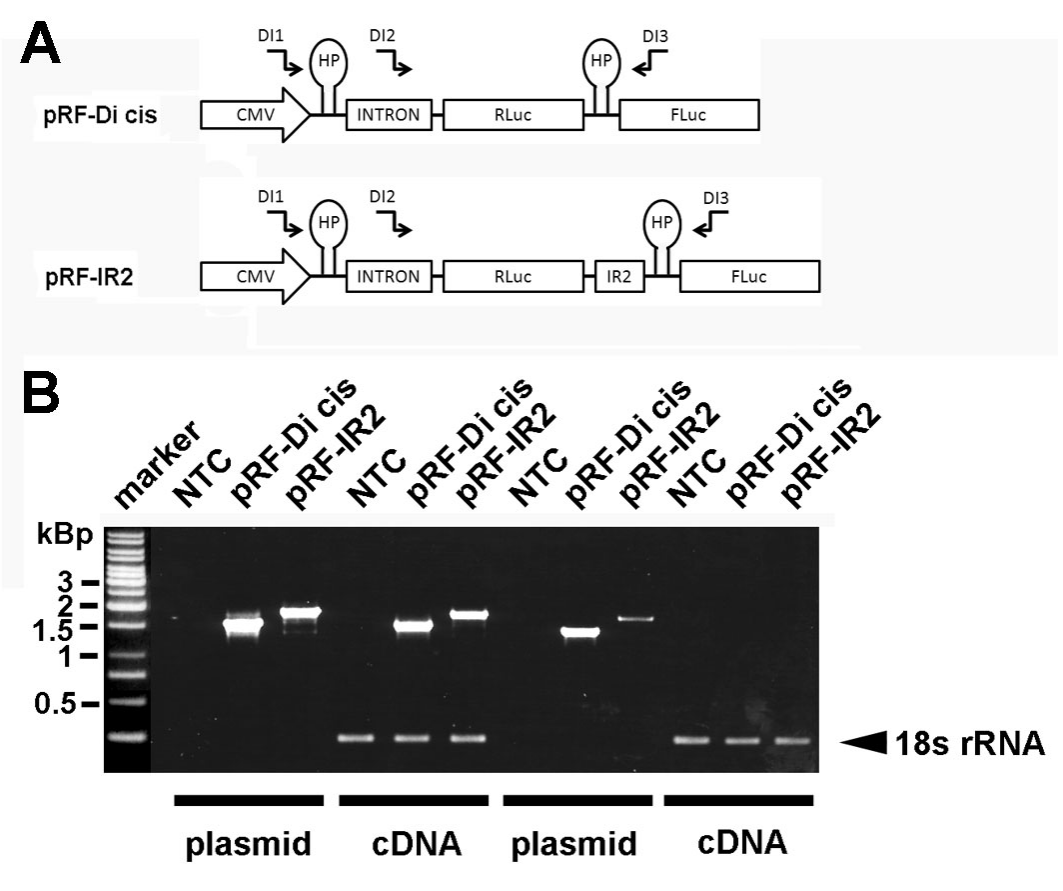
**RT-PCR analysis of Di-cis constructs**. (A) Schematic view of the Di-cis constructs used for the RT-PCR analysis including the relative position of the haipin structure (HP), the chimeric human globin intron (137 nt), and the primers used for RT-PCR analysis indicated as arrows (see Additional file 5). B) RT-PCR from N2A cells transiently transfected with the control vector pRF-Di cis and the pRF-IR2 construct. A 1 kbp DNA ladder is shown on the left side of the gel. NTC, denotes no template control, PCR products generated from plasmid DNA are denoted as plasmid. RT-PCR products are indicated as cDNA. The 18s rRNA control PCR served as internal standard as described previously (49)

Splice variants which may have evaded northern blot detection (data not shown) due to the lower sensitivity were excluded by RT-PCR. The PCR strategy is outlined in (Fig. 7). RT-PCR with the cDNA from the control Di-cis vector pRF-Di cis or the IR2 containing vector pRF-IR2 with the primer pair DI1/DI3 generated single amplicons of ~1,3 and ~1,6 kBp. The primer DI1 is located immediately adjacent to the CMV promoter/enhancer element and 5' upstream of the chimeric intron and the hairpin structure included in the vector. The removal of the chimeric intron (138 bp) from the messages is documented by RT-PCR using the primer DI2 in combination with the primer DI3. As expected no amplification product was generated.

Next we performed western blot analysis of N2A cells expressing the control vector pRE-Dis cis or pRE-IR2 (see Additional file 2). We assumed that a cryptic promoter activity should lead to a reduced RLuc protein and increased EGFP protein expression when EGFP is translated from a monocistronic message. In contrast, when both proteins are generated from a single mRNA we expected that RLuc expression should be unaffected but EGFP expression substantially increased in the presence of the IR2 element. As shown, simultaneous detection of the *Renilla *luciferase and EGFP showed that the translation of the second cistron was greatly enhanced when the IR2 element was included in the bicistronic vector. In line with the latter assumption the expression of the first cistron appeared unchanged.

In summary, the results obtained by independent methods conclusively suggest that p11-CT is not expressed from a monocistronic mRNA.

### The p11-CT product is partially located in the nucleus in vitro

**Figure 8:**
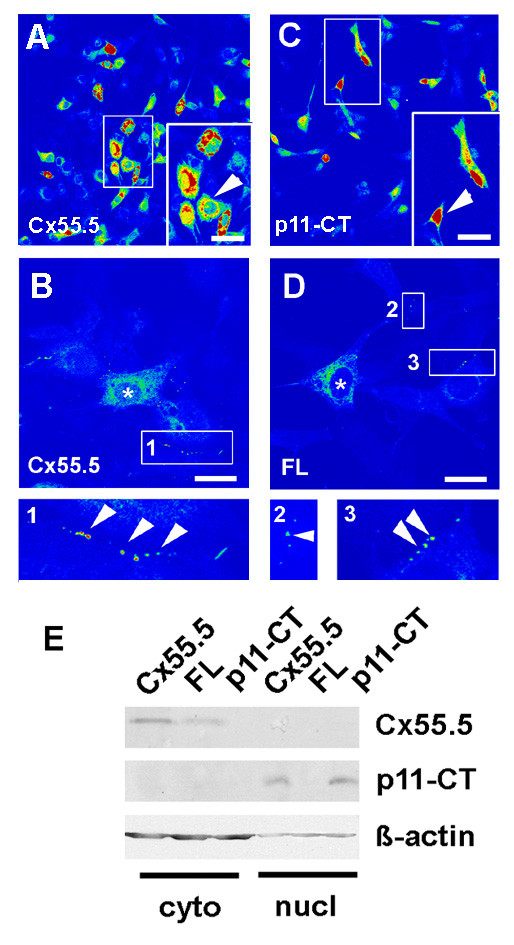
**Subcellular localization of the Cx55.5 protein isoforms in vitro**. The subcellular distribution of FLAG tagged Cx55.5, FL and p11-CT proteins was determined by immunocytochemistry. All constructs were transiently expressed in NIH3T3 cells. Confocal images were collected using the LSM510 Meta system and software. Pictures shown represent single optical planes which are presented in pseudocolours for better representation of the staining intensity. Red label indicates maximal concentration. A, B) Wild-type Cx55.5 construct (WT) co-expressing the full length Cx55.5-FLAG and p11-CT-FLAG fusion proteins. (magnification in A: 40×, Scale bar = 30 μm; in B: 63× magnification, Scale bar = 15 μm). Highest concentrations occur in perinuclear regions and the nucleus. B) Higher magnification of the WT construct with a ROI (1) showing punctuate membrane label. C) FLAG-tagged p11-CT with maximal concentration in the nuclei. The arrowheads in the inset indicates the characteristic nuclear localization of p11-CT. (magnification: 40×; Scale bar 30 μm). D) FLAG-tagged FL protein encoding for the full length Cx55.5 protein only (magnification 63×, Scale bar: 15 μm). Besides lack of nuclear staining plaque-like protein assemblies occur at sites of cell contacts (indicated by arrowheads in inset 2 and 3). Such membrane bound fluorescence was not observed with the p11-CT construct. (E) Western blot analysis of cytosolic and nuclear fractions of Cx55.5-FLAG, FL-FLAG and p11-CT-FLAG transfected N2A cells. 20 μg of total nuclear and 10 μg of cytosolic protein was separated by SDS-PAGE on a 12% gel. Immunoblot detection was done using anti-Cx55.5 as primary antibody (1 μg/ml). The upper lanes represent the full length Cx55.5 protein products expressed by the Cx55.5 wildtype protein and the FL mutant. The middle lane refers to the p11-CT protein expressed by the Cx55.5 wildtype and p11-CT proteins, but not by the FL mutation. The lower blot depicts the β-actin control. Note that the β-actin signal in the nuclear fraction is significantly reduced despite the fact that more protein was loaded on the gel. This indicates a minimal contamination with cytoplasmic remnants.

We further addressed the question whether the p11-CT protein showed a subcellular localization *in vitro *comparable with the *in vivo *results. In these experiments the large EGFP-tag (27 kDa) was replaced in all constructs by the 8 amino acid FLAG-epitope (DYKDDDDK; ~1 kDa) in order to exclude the possibility that EGFP might influence the subcellular localization. We analyzed the distribution of the FLAG tagged Cx55.5 (WT), FL (ATG at nt1201 to GCG) and p11-CT protein (frame shift mutation at nt1179 in the Cx55.5 coding region) products by immunocytochemistry using an antibody specific for the FLAG epitope [28]. The confocal images show that in cells transfected with full length Cx55.5 staining was most abundant in the perinuclear areas with a reduced but consistent staining of most nuclei (Fig. 8A). At higher magnification this distribution was confirmed (enlarged inset in Fig. 8A). In addition small plaque-like structures were detected at the sites of cell contacts suggesting that FLAG-tagged Cx55.5 is capable to form junctions between transfected cells (Fig. 8B, inset 1). Transiently transfected NIH3T3 cells expressing p11-CT alone showed a protein distribution distinct from cells expressing full length Cx55.5. Here, p11-CT was also found both in the cytoplasm and nucleus, however, the nuclear localization was substantially pronounced when compared to the full length Cx55.5 (Fig. 8C, enlarged inset). This experimental finding is consistent with *in silicio *analyses which predict a potential nuclear localization of p11-CT on the basis of the amino acid composition, physiochemical properties, dipeptide composition and PSI-BLAST using the subcellular protein localization prediction softwares *pSLIP *and *ESLpred *[[29,30] data not shown]. Finally, we observed that the FL-mutant of Cx55.5 lacking the p11-CT start site and thus encoding for the full length protein only was found in the perinuclear area and plasmamembrane forming plaque like structures at the cell periphery (Fig. 8D, insets 2 and 3). In contrast to Cx55.5 and p11-CT, nuclei were devoid of nuclear staining. Next, western blots using nuclear and cytoplasmic extracts prepared from Cx55.5-FLAG, FL-FLAG and p11-CT-FLAG transfected N2A cells were performed. N2A cells were selected for this experiment in favour of the NIH3T3 cells shown above due to a higher ratio of nucleus to cytoplasm. This experiment confirmed that the full length Cx55.5 and FL proteins were enriched in the cytosolic protein fraction which includes membrane proteins (Fig. 8E). In the experiment shown no p11-CT protein was detected in this protein fraction. In contrast, a band of ~16 kDa translated from the Cx55.5 and p11-CT transfected cells was substantially enriched in the nuclear fraction consistent with our previous observations. The subcellular localization of Cx55.5, FL and p11-CT proteins were consistent with initial experiments performed with the EGFP-tagged protein variants (see Additional file 3) demonstrating that the two different protein tags used in this study had a neglectable impact on the subcellular localization of the Cx55.5 isoforms.

In summary, we provide evidence that an IRES-mediated molecular process may account for the generation of a small CT domain of the HC connexin Cx55.5, and that this protein product is potentially capable to translocate into the nucleus both *in vivo *and *in vitro*.

## Discussion and Conclusion

In this report, we describe that the internal translation involving a putative IRES element located in the coding region of connexin Cx55.5 leads to the formation of two alternative protein products *in vivo *and *in vitro*. Our initial observation of low levels of p11-CT immunoreactivity inside the nuclei of horizontal cells *in vivo *has led us analyze putative molecular mechanisms underlying the generation of this Cx55 protein fragment.

By performing sequence analyses of the coding region of Cx55.5 we found several in frame ATG codons. Three candidate in-frame ATG codons in the long carboxy-terminal tail of Cx55.5 were taken into consideration. The mutation analysis confirmed the ATG at nucleotide position 1201 as translation initiation site giving rise to a protein product with a theoretical molecular weight of 11 kDa in case of internal translation.

On the basis of the above observations we hypothesized that the generation of the p11-CT is achieved by an IRES element in the coding region of Cx55.5. Since IRES mediated activities have been scrutinized recently [31,32] we performed stringent control experiments to rule out activities of cryptic promoters, RNAse cleavage or cryptic splicing. To date the number of IRES elements found in connexin genes is limited and those IRES elements identified are restricted to the 5'UTR of Cx43, Cx32 and Cx26 [24-26]. More elements have been reported in the 5'UTR of other eukaryotic genes [33-38]. The existence of IRES elements in the coding region of eukaryotic genes is still a rare observation with only a few reports in the literature [39,40], where in some examples similarly to the p11-CT the carboxy-terminal domain has been described to be internally translated [41].

Supporting data on a nuclear presence of p11-CT come from our *in vitro *studies which included confocal laser imaging and western blots of nuclear extracts. Both demonstrated nuclear localization of the p11-CT fusion protein when transfected in NIH3T3 cells. Different from the transfected cells the abundance is less under *in situ *conditions and restricted to a subpopulation of horizontal cells. This, however, is as expected, since the amount of physiologically expressed and hence translocated protein must be regarded to be lower in the intact retina as compared to the over-expressing cell lines.

A nuclear localization of a carboxy-terminal domain of a connexin has been already reported in case of Cx43 [22]. A nuclear translocation of carboxy-terminal domains would be of paramount importance as far as gap junction biology is concerned. The existence of a molecular mechanism initiating an internal expression of CT fragments could provide first insight into connexin properties not readily explainable by its channel forming properties. A separate expression of biologically active CT-domains and their nuclear translocation can endow gap junction proteins with the capability of modulating gene expression directly in response to changes in physiological activities and/or pathophysiological challenges.

The biological significance of a separate expression of a CT-domain of Cx55.5 via IRES mediated internal translation is still to be established. An argument in favour of a possible functional link between expression of Cx55.5 and retinal activity derives from the observation that both Cx55.5 isoforms are strictly expressed in HCs. Most importantly, the Cx55.5 full length protein is known to accumulate exactly at those sites known to undergo a dramatic light dependent reorganization of the postsynaptic compartment of the HC/RC/BPC complex in form of reshaping of the HC dendritic spinules after light withdrawal [12,42-44]. Since these sites have been proven to be responsible for changes of functional responsiveness of the HC/RC/BPC complex accompanying dark adaptation [44], a signalling pathway involving Cx55.5 is suggestive. At present the molecular mechanism involved is unclear but our recent identification of the RNA binding polypyrimidine tract binding protein (PTB) as interaction partner at the putative Cx55.5 IRES element (M.U-H, unpublished data) is suggestive for a mechanism involving the cAMP/PKA signalling pathway [45]. Furthermore, the basic properties of the p11-CT domain with a calculated pI of >10 may resemble properties comparable to the basic DNA binding domains of some transcription factor gene families [46]. Therefore, future studies need to uncover whether the subcellular localization and/or potential DNA binding capabilities of p11-CT underlay mechanisms similar to C/EBPdelta [47] or Gli1 [48], two nuclear transcription factors known to shuttle between cytoplasm and nucleus upon activation of a PKA-driven pathway.

The described activity of an IRES sequence and the translation of the p11-CT fragment of Cx55.5 may resemble a novel regulative mechanism which governs the plasticity of the PRC/HC/BC complex. It awaits further exploration to unearth the mechanistic background which links the functional with the molecular side.

## Methods

### Animals

Zebrafish were kept at 28°C in aerated tanks filled with tap water circulating through an bacterial filter systems. The fish were kept on a 14 hour ON/10 hour OFF light-dark cycle. All animal experiments were carried out according to the guidelines of the German Animal Protection Law in its present version (1998) and under the responsibility of the ethical committee of the Royal Netherlands Academy of Arts and Sciences acting in accordance with the European Communities Council Directive of 24th November 1986 (86/609/EEC). All experiments including animals were kept to the necessary minimum.

### Plasmid constructs

Full length Cx55.5 (nt1–nt1497) was obtained by PCR amplification of the coding region from a genomic clone (GI:77747480) using primer pair S1 and AS1 (Additional File 5). The PCR product was ligated in-frame into pEGFP-N3 (BD Biosciences Clontech, CA, USA) and sequence confirmed. All other plasmid constructions summarized in the Additional file 4 derived from this construct and were cloned using standard recombinant DNA techniques. The Di-cistronic vector pRF-Di cis comprising the *Renilla *luciferase as first cistron, the *Firefly *luciferase as second cistron, the human globin gene intron and stable hairpin structures has been described [25]. The plasmid was a kind gift from Dr. Rudolf Werner (Department of Biochemistry and Molecular Biology, University of Miami, School of Medicine). The pGL3-control vector was obtained from Promega (Promega Corporation, Madison, WI, USA).

### Cell culture, transfection and reporter-assays

HeLa, NIH3T3 and N2A cells were purchased and maintained in cell culture as recommended by the ATCC (LGC Promochem GmbH, Wesel Germany). For determination of IRES activity, 2 × 10^4 ^N2A cells were plated in 96 well flat bottom plates (BD Biosciences), transiently transfected using 100 ng plasmid DNA and the Effectene^® ^transfection protocol (Qiagen, Hilden, Germany). 48 hours after transfection, luciferase activity was measured in an Orion II Micro plate Luminometer (Berthold Detection Systems, Pforzhein, Germany), using the Dual-Luciferase Reporter assay system (Promega Corp., Madison, Wisconsin, USA). IRES activity was expressed as the ratio of *Firefly *luciferase/*Renilla *luciferase (FLuc/RLuc) with the activity of the control vector (pRF Di-cis.) set to 1. Each experiment was performed 5 times with all constructs in triplicates. Data are expressed as mean ± SEM.

### Generation of a Cx55.5 specific polyclonal antibody

A polyclonal Cx55.5 antibody was generated by immunization of Chinchilla rabbits. The antibody was generated against the carboxy-terminal domain of Cx55.5 (amino acids 231–498) which was expressed as a GST fusion protein in the E. coli strain BL21. The serum was affinity purified using GST-Cx55.5 crosslinked to a HITrap-Sepharose column matrix and eluted as recommended by the manufacturer (GE Healthcare UK Ltd, Little Chalfont Buckinghamshire, UK). The specificity of the antibody was confirmed by western blotting and immunocytochemistry using preimmune serum and/or preabsorption with GST-Cx55.5 as controls. Cross reactivity with the second horizontal cell connexin (Cx52.6) was excluded by absorption to GST-Cx52.6 as described [12].

### Western blot analyses

For western blot analyses of transfected cell lines, 2 × 10^5 ^N2A or NIH3T3 cells were grown in 12 well plates (BD Biosciences). Transient transfections were performed using 300 ng plasmid DNA and the Effectene^® ^transfection protocol (Qiagen). 48 hours after transfection, cytosolic and nuclear extracts were prepared according to the manufactures protocol (Active Motif Nuclear Extraction Kit, Rixensart, Belgium). 20 μg of each protein fraction was separated on 10% SDS PAGE and processed as described previously [49]. In some experiments the detection antibody was replaced and the IRDye 680 Goat Anti-Mouse IgG detection antibody (1:20,000) used in combination with the Odyssey infrared detection system (LI-COR Biosciences, St. Lincoln, NE, USA).

For western blot analysis of adult zebrafish tissues total protein extracts were isolated from freshly dissected retina and brain by direct homogenization in denaturing Leammli buffer. Alternatively, cytosolic and nuclear protein fractions were generated with the identical kit described above. 20 μg protein was separated by 15% SDS-PAGE, transferred to 0.2 μm NC membrane (Protran BA83, Schleicher & Schüll BioScience, Dassel, Germany) and processed as described above. Primary antibodies were diluted 1:2,000 (anti-GFP; Roche Applied Science, Mannheim, Germany), 1:1,000 (anti-FLAG; Sigma-Aldrich, St. Louis, USA) and 1:7,500 (anti-β actin; Sigma-Aldrich). The primary anti-Cx55.5 antibody was used at 1 μg/μl.

### RNA expression analysis

For expression analysis, 2 × 10^6 ^N2A cells were seeded in 6-well plates (BD Biosciences). RT-PCR was performed as a test for mRNA splicing in N2A cells following DNA transfection of the control Di-cis vector and the IRES containing Di-cis constructs. Total RNA was isolated as described above from transiently transfected N2A cells. First strand cDNA synthesis was carried out as described [17] and PCR amplified using the primer pairs DI1/DI3 and DI2//DI3 (see Additional file 5).

### Immunohistochemistry and immunoelectronmicroscopy

Eyes were isolated from cervically transected fish and processed for immunohistochemistry and immuno electron microscopy as described [12]. Confocal image analysis was performed using the LSM 510 Meta system (Zeiss), equipped with argon and HeNe lasers, 40× (NA 1.4) and 63× (NA 1.4) oil objectives and the LSM 510 Meta software as described [49].

## Authors' contributions

MU–H conceived the study, carried out the molecular genetic studies and drafted the manuscript. GZ participated in the molecular genetic studies, performed the confocal imaging experiments, generated the Cx55.5 antibody and helped to draft the manuscript. JK and MK carried out the ImmunoEM and participated in protein analysis, RD participated in its design and coordination and helped to draft the manuscript. All authors read and approved the final manuscript.

## Supplementary Material

Additional file 1 **Cx55.5 protein localization in the outer retina of the zebrafish**. (A) Overview of the retina demonstrating the highly restricted expression of Cx55.5 in a single band like cell layer. (B) The higher magnification demonstrates the co-localization of Cx55.5 and GluR2 proteins in horizontal cells. (Abbreviations: ganglion cell layer, GCL; inner plexiform layer, IPL; inner nuclear layer, INL; outer plexiform layer, OPL; outer nuclear layer, ONL; pigment epithelium, PE). Scale bar in Fig A = 20 μm. Scale bar in B = 10 μm. Click here for file

Additional file 2 **IR2 mediated upregulation of GFP expression in the bi-cistronic vector assay**. Western blot of pRE and pRE-IR2 transfected N2A cells. This experiment demonstrates that the IRES element substantially promoted expression of the EGFP protein. Click here for file

Additional file 3 **Subcellular localization of Cx55.5 protein variants in transfected NIH3T3 cells**: Confocal laser scanning imaging of the subcellular distribution of EGFP and the Cx55.5-EGFP fusion protein constructs in NIH3T3 cells. This experiment confirmed the distinct distribution of FL and p11-CT isoforms. Note that p11-CT showed a pronounced nuclear accumulation with some staining in the cytoplasm. Scale bar: 25 μm. Click here for file

Additional file 5 Summary of PCR primers used for plasmid construction, mutagenesis and PCR. This table summarizes all primers used in this study. Click here for file

Additional file 4 Summary of Cx55.5 plasmid constructs. This table summarizes all plasmid constructs used in this study. Click here for file
