# Supplementary figures and images for: IRES-mediated translation of the carboxy-terminal domain of the horizontal cell specific connexin Cx55.5 in vivo and in vitro
Source: BMC Mol Biol. 2008 May 27;9:52. doi: 10.1186/1471-2199-9-52 (PMC2435236; doi:10.1186/1471-2199-9-52)

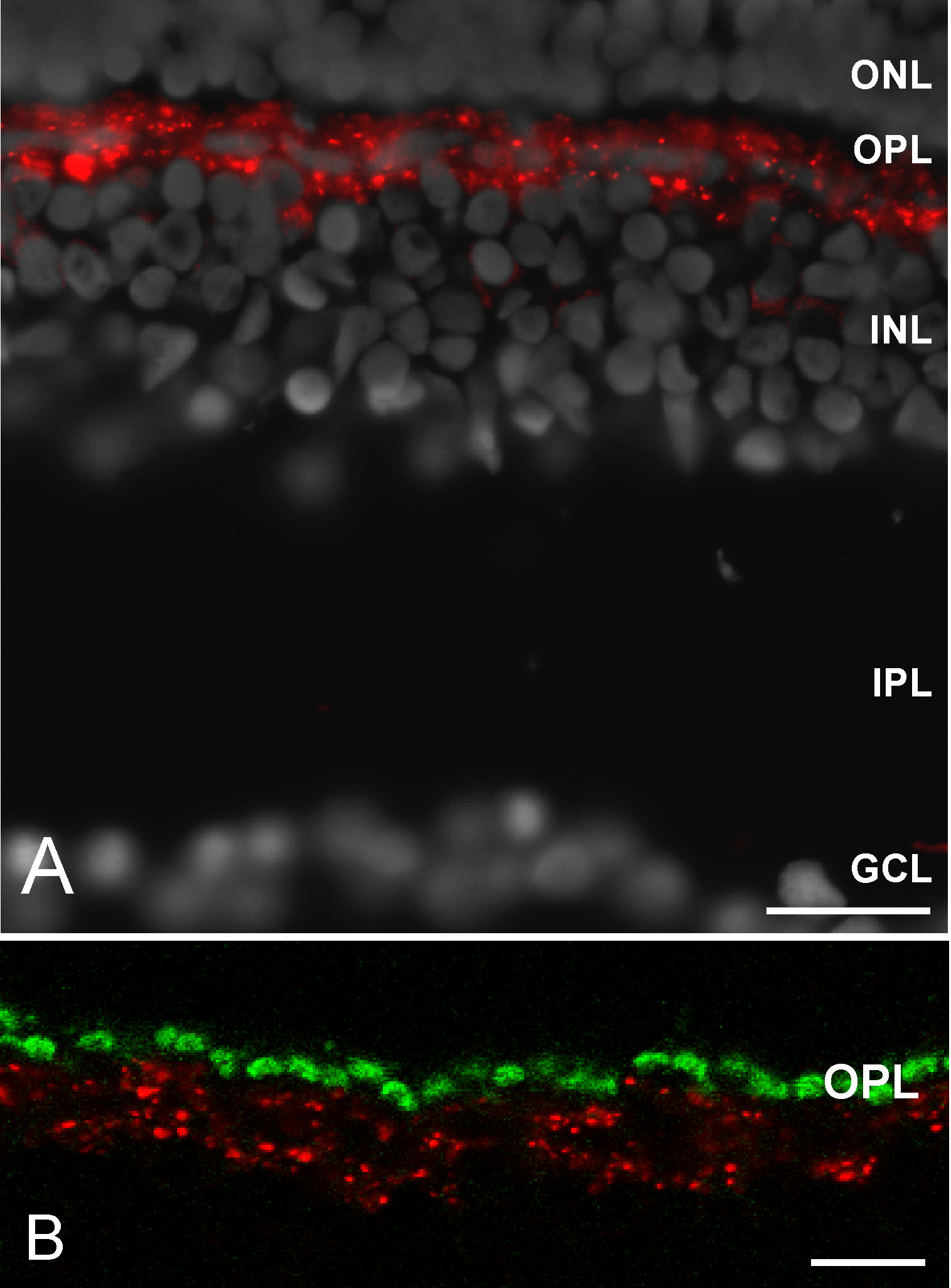

Supplement: Additional file 1 — Cx55.5 protein localization in the outer retina of the zebrafish. (A) Overview of the retina demonstrating the highly restricted expression of Cx55.5 in a single band like cell layer. (B) The higher magnification demonstrates the co-localization of Cx55.5 and GluR2 proteins in horizontal cells. (Abbreviations: ganglion cell layer, GCL; inner plexiform layer, IPL; inner nuclear layer, INL; outer plexiform layer, OPL; outer nuclear layer, ONL; pigment epithelium, PE). Scale bar in Fig A = 20 μm. Scale bar in B = 10 μm. [file 1471-2199-9-52-S1.jpeg]

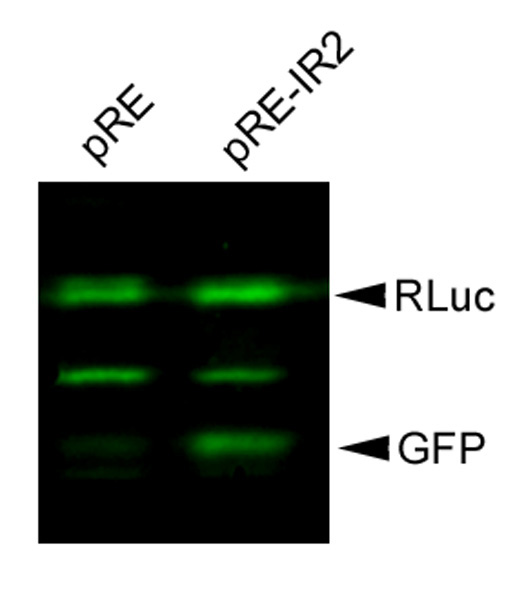

Supplement: Additional file 2 — IR2 mediated upregulation of GFP expression in the bi-cistronic vector assay. Western blot of pRE and pRE-IR2 transfected N2A cells. This experiment demonstrates that the IRES element substantially promoted expression of the EGFP protein. [file 1471-2199-9-52-S2.jpeg]

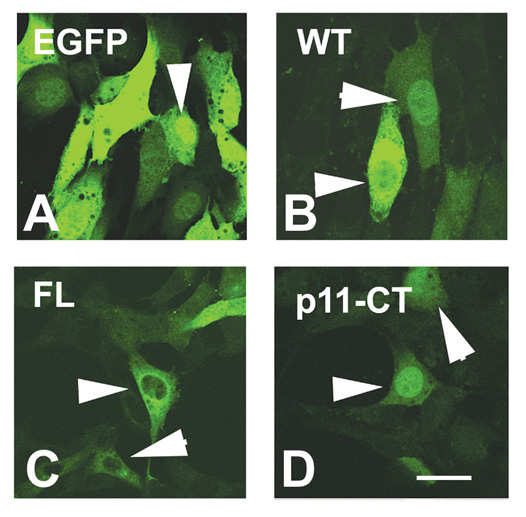

Supplement: Additional file 3 — Subcellular localization of Cx55.5 protein variants in transfected NIH3T3 cells: Confocal laser scanning imaging of the subcellular distribution of EGFP and the Cx55.5-EGFP fusion protein constructs in NIH3T3 cells. This experiment confirmed the distinct distribution of FL and p11-CT isoforms. Note that p11-CT showed a pronounced nuclear accumulation with some staining in the cytoplasm. Scale bar: 25 μm. [file 1471-2199-9-52-S3.jpeg]
