# Supplementary material for: IRES-mediated translation of the carboxy-terminal domain of the horizontal cell specific connexin Cx55.5 in vivo and in vitro
Source: BMC Mol Biol. 2008 May 27;9:52. doi: 10.1186/1471-2199-9-52 (PMC2435236; doi:10.1186/1471-2199-9-52)
Supplement: Additional file 5 — Summary of PCR primers used for plasmid construction, mutagenesis and PCR. This table summarizes all primers used in this study. [file 1471-2199-9-52-S5.doc]

**Additional file 5:** Summary of PCR primers used for plasmid construction, mutagenesis and PCR

| primer | application | comment | sequence |
| --- | --- | --- | --- |
| S1 | PCR cloning | EcoRI-site | 5’-CCG *GAA TTC* GTT CAT GTT TCT TTC TTC TTA-3` |
| S2 | PCR cloning |  | 5’-TCT TCA TGG TGT TCA TGC AAT GC- 3’ |
| S3 | PCR cloning |  | 5-CCA GCA TGG TAA AGA AAC CTT GG-3’ |
| S4 | PCR cloning. | FLAG oligo | 5´- GAT CAG ATT ACA AGG ATG ACG ACG ATA AGT AG-3 |
| S5 | PCR cloning | EcoRI-site | 5`-CCG *GAA TTC* TTC ATG GTG TTC ATG CAA-3` |
| DI1 | PCR |  | 5´- gct tct gac aca aca gtc tcg aac tta ag -3´ |
| DI2 | PCR |  | 5´- gac caa tag aaa ctg ggc ttg tcg aga cag - 3´ |
| DI3 | PCR |  | 5´- ctt cca gcg gat aga atg gcg ccg ggc c - 3´ |
| AS1 | PCR cloning | BamHI-site | 5’-ATC *GGA TCC* AAT TTG TAA GTG TGT GGG AGC -3’ |
| AS2 | PCR cloning | FLAG oligo | 5´ GGC CCT ACT TAT CGT CGT CAT CCT TGT AAT CT-3´ |
| AS3 | PCR cloning | XhoI | 5`-CCG *CTC GAG* GCT GGA TAA GGC ATG-3` |
| M1 | mutagenesis | ATG 🡪 GCG | 5´-CTC ATC CAG C*GC G*GT AAA GAA ACC-3 |
| M2 | mutagenesis | frameshift mutation | 5´-CAC ACC AGA GAA *T*TC ATC TCA TGC CTC-3´ |

Note: restriction sites introduced to facilitate cloning are in italics and underlined.
