# Supplementary material for: IRES-mediated translation of the carboxy-terminal domain of the horizontal cell specific connexin Cx55.5 in vivo and in vitro
Source: BMC Mol Biol. 2008 May 27;9:52. doi: 10.1186/1471-2199-9-52 (PMC2435236; doi:10.1186/1471-2199-9-52)
Supplement: Additional file 4 — Summary of Cx55.5 plasmid constructs. This table summarizes all plasmid constructs used in this study. [file 1471-2199-9-52-S4.doc]

**Additional file 4:** Summary of Cx55.5 plasmid constructs

| name | basic plasmid | Cx55.5 insert (nt) | modification | comment |
| --- | --- | --- | --- | --- |
| WT | pEGFP-N3* | 1-1497 | none | wild type |
| WT-FLAG | pEGFP-N3 | 1-1497 | EGFP 🡪 FLAG | TAG replaced |
| CT-629 | pEGFP-N3 | 629-1497 | N-terminal deletion | full length CT |
| CT-1196 | pEGFP-N3 | 1196-1497 | N-terminal deletion | truncated CT |
| FL | pEGFP-N3 | 1-1497 | ATG 🡪 GCG | mutation (nt1201-nt1202) |
| FL-FLAG | pEGFP-N3 | 1-1497 | EGFP 🡪 FLAG | TAG replaced |
| p11-CT | pEGFP-N3 | 1-1498 | T | insertion (nt1179) |
| p11-CT-FLAG | pEGFP-N3 | 629-1497 | EGFP 🡪 FLAG | TAG replaced |
| pRF-IR1 | pRF-Di cis** | 631-1200 | none | putative IRES-element |
| pRF-IR2 | pRF-Di cis | 631-990 | none | putative IRES-element |
| pRE-IR1 | pRF-Di cis | 631-1200 | Fluc 🡪 EGFP | reporter replaced |
| pRE-IR2 | pRF-Di cis | 631-990 | Fluc 🡪 EGFP | reporter replaced |
| pRF-IR1ΔCMV | pRF-Di cis | 631-1200 | ΔCMV | promoterless |
| pRF-IR2ΔCMV | pRF-Di cis | 631-990 | ΔCMV | promoterless |
| pRE-IR2ΔCMV | pRF-Di cis | 631-990 | Fluc 🡪 EGFP; ΔCMV | reporter replaced; promoterless |

* pEGFP-N3 was obtained from BD Biosciences Clontech, CA, USA

** pRF-Di cis was a gift of Dr. Rudolf Werner (Department of Biochemistry and Molecular Biology, University of Miami, School of Medicine).
